# Using the Internet to Train Therapists: Randomized Comparison of Two Scalable Methods

**DOI:** 10.2196/jmir.8336

**Published:** 2017-10-18

**Authors:** Zafra Cooper, Suzanne Bailey-Straebler, Katy E Morgan, Marianne E O'Connor, Caroline Caddy, Layla Hamadi, Christopher G Fairburn

**Affiliations:** ^1^ Department of Psychiatry University of Oxford Oxford United Kingdom; ^2^ Department of Psychiatry Yale School of Medicine New Haven, CT United States; ^3^ London School of Hygiene and Tropical Medicine London United Kingdom

**Keywords:** psychotherapy, training, Internet, eating disorders, cognitive therapy

## Abstract

**Background:**

One of the major barriers to the dissemination and implementation of psychological treatments is the scarcity of suitably trained therapists. The currently accepted method of training is not scalable. Recently, a scalable form of training, Web-centered training, has been shown to have promise.

**Objective:**

The goal of our research was to conduct a randomized comparison of the relative effects of independent and supported Web-centered training on therapist competence and investigate the persistence of the effects.

**Methods:**

Eligible therapists were recruited from across the United States and Canada. They were randomly assigned to 1 of 2 forms of training in enhanced cognitive behavior therapy (CBT-E), a multicomponent evidence-based psychological treatment for any form of eating disorder. Independent training was undertaken autonomously, while supported training was accompanied by support from a nonspecialist worker. Therapist competence was assessed using a validated competence measure before training, after 20 weeks of training, and 6 months after the completion of training.

**Results:**

A total of 160 therapists expressed interest in the study, and 156 (97.5%) were randomized to the 2 forms of training (81 to supported training and 75 to independent training). Mixed effects analysis showed an increase in competence scores in both groups. There was no difference between the 2 forms of training, with mean difference for the supported versus independent group being –0.06 (95% Cl –1.29 to 1.16, *P*=.92). A total of 58 participants (58/114, 50.9%) scored above the competence threshold; three-quarters (43/58, 74%) had not met this threshold before training. There was no difference between the 2 groups in the odds of scoring over the competence threshold (odds ratio [OR] 1.02, 95% CI 0.52 to 1.99; *P*=.96). At follow-up, there was no significant difference between the 2 training groups (mean difference 0.19, 95% Cl –1.27 to 1.66, *P*=.80). Overall, change in competence score from end of training to follow-up was not significant (mean difference –0.70, 95% CI –1.52 to 0.11, *P*=.09). There was also no difference at follow-up between the training groups in the odds of scoring over the competence threshold (OR 0.95, 95% Cl 0.34 to 2.62; *P*=.92).

**Conclusions:**

Web-centered training was equally effective whether undertaken independently or accompanied by support, and its effects were sustained. The independent form of Web-centered training is particularly attractive as it provides a means of training large numbers of geographically dispersed therapists at low cost, thereby overcoming several obstacles to the widespread dissemination of psychological treatments.

## Introduction

Psychological treatments are difficult to disseminate [1,2]. One of the major barriers to their dissemination and implementation is the scarcity of suitably trained therapists [3]. The currently accepted method of training typically involves attending a specialist workshop, reading relevant texts, and receiving subsequent supervision from someone expert in the treatment [4]. A fundamental flaw with this method is that it is not scalable, as it is both labor-intensive and costly [5,6].

One solution to the problem of scalability is the “train the trainer” model in which an expert provides training to an individual who subsequently trains other providers, thereby increasing the reach of the training [7,8]. While this method has advantages in comparison to the conventional method [3], it is still relatively resource-intensive and potentially slow to have an impact [9].

Recently there has been growing interest in training therapists using the Internet [10]. This has a number of potential advantages. Training can be offered simultaneously to large numbers of geographically dispersed trainees with Web resources that can be accessed at any time and from any place. Furthermore, trainees can review and revisit material in a way that potentially reinforces learning and prevents subsequent therapist drift [9,11]. In addition, clinical illustrations and formative assessments such as knowledge tests can be integrated into the training program. The program can also be updated regularly to incorporate new information. Last, data collection on website usage can provide information to inform and improve the training process.

We have developed a form of therapist training called Web-centered training [12,13]. It differs from conventional training in that the training is fully automated with the expertise residing within the program rather than provided by an outside expert. Thus, Web-centered training can be undertaken completely autonomously (independent training). Alternatively, it can be accompanied by support from a nonspecialist worker (supported training), an approach that closely resembles supported or guided self-help [14-16], with the aim of the support being to increase adherence to the training program. As the role of the support worker is solely to encourage the trainee to follow the training program rather than to provide clinical supervision, it can be undertaken by people with limited training. Thus, the supported form of Web-centered training is also scalable. A recent proof-of-concept study of this supported form of Web-centered training found that the method was feasible and acceptable to therapists and was effective in improving therapist competence [13]. This finding requires replication. In addition, whether supported training is more beneficial than undertaking training independently needs to be investigated. A further question concerns the persistence of the benefits of training, as transitory effects would be of limited value.

Our study had 2 aims. The first was to determine the relative effects of independent and supported Web-centered training on therapist competence, and the second was to investigate the persistence of these effects.

## Methods

### Design

The study was a randomized comparison of 2 educational interventions, independent and supported Web-centered training. Eligible therapists were randomly assigned to 1 of these 2 forms of training. Therapist competence was assessed before the training, after 20 weeks of training, and at 6 months after the completion of training.

The research protocol was submitted to the Oxford University Central Research Ethics Committee. As the intervention was judged to be educational rather than clinical, the committee decided that formal ethical approval was not required.

### Recruitment

Participants were recruited from across the United States and Canada by advertisements offering free training in enhanced cognitive behavior therapy (CBT-E), a multicomponent evidence-based psychological treatment for any form of eating disorder [17,18]. Potential participants had to be licensed mental health professionals who were prepared to take part in research evaluating Web-centered training. Advertisements were placed in the publications of the following professional bodies: American Psychological Society, National Association of Social Workers, American Psychiatric Nurses Association, American Psychiatric Association, and Academy for Eating Disorders. These advertisements included a link to an online description of the training and study.

Participants had to meet the following eligibility criteria: have been previously trained in delivering short-term psychological treatments, work with individuals with eating disorders, be willing to be randomized to independent or supported Web-centered training, be willing to devote at least 9 hours to the training program, be able to treat 1 or more patients using CBT-E during the 20-week period of training, and provide informed digital consent. In the information provided to participants, it was stressed that clinical responsibility for their patients would remain with their local clinical team and not be shared with the researchers or support workers.

Eligible participants were asked to complete a brief online survey about their professional background, age, gender, and clinical experience. They also completed an online therapist competence assessment. They were subsequently sent a link to the training website together with instructions about how to use the training program, tailored to whether they were to receive independent or supported training. In addition, they were sent brief details about the minimum technical specifications for accessing the website.

### Training Program

The CBT-E Web-centered training program has 2 main parts: the Course and the Library. A summary of the content of the training is provided in Multimedia Appendix 1; the CBT-E training program and complete details are provided elsewhere [13]. Briefly, the Course is linear in nature and takes between 8 and 9 hours to complete. It is a detailed practical description of how to implement the main focused form of CBT-E given by an expert on the treatment (CGF). This description is delivered in the form of multiple brief video presentations accompanied by handouts and interspersed with formative learning exercises, video recordings of acted illustrations of the treatment, and tests of knowledge together with feedback. While working through the Course, trainees are encouraged to read relevant sections from the treatment manual [19] and treat 1 or 2 patients.

The second part of the training website, the Library, contains all the material in the Course including the handouts, learning exercises, and clinical illustrations in indexed form as well as further longer clinical illustrations. In addition, there is a large amount of supplementary material on how to use CBT-E with specific subgroups of patients including those who are severely underweight and those with clinical perfectionism, core low self-esteem, and marked interpersonal difficulties. There is also a detailed account of how to use CBT-E to treat younger patients.

The participants were granted access to the Course and the core Library material from the start of training, and they continued to have access during the follow-up. They only had access to the supplementary Library material focusing on specific subgroups of patients once they had completed the study.

Information about participants’ use of the training program, in particular the number of Course modules viewed and completed, was obtained from the website.

Participants randomized to independent training were given access to the Course and the Library. There was no external support, but they did receive reminder emails at 6, 10, 14, and 18 weeks informing them of the number of weeks of training that had elapsed and the number of weeks remaining.

Participants randomized to supported training were offered up to 12 telephone calls over the 20-week period of training, each lasting no longer than 30 minutes. These were weekly for the first 4 weeks and then every other week. The calls were designed to be purely supportive in nature. Their goal was to encourage participants to work through the training material and implement CBT-E with their training cases [13]. A protocol defined the nature and limits of the supportive role. The support was provided by research assistants who were not clinicians and had no experience delivering CBT-E. They were supervised by 2 senior clinicians (ZC and SBS).

### Assessment

Participants’ competence at delivering CBT-E was assessed before training, immediately following training, and 6 months later. It was measured using a scalable online measure with sound psychometric properties that had previously been developed independently of the creation of the training website. Its development and validation are described in detail in a separate publication [20]. Measure development included detailed blueprinting, state-of-the-art item writing, independent item review, and initial field-testing, followed by formal Rasch analysis to test for good model fit. Strict criteria of unidimensionality were met by stepwise exclusion of misfitting items until there was no individual item misfitting at *P*<.01. The resulting measure consists of 22 items addressing trainee knowledge and understanding of CBT-E and its implementation (ie, applied knowledge). The instrument generates a total score (out of a possible 22), and trainees can be classified as scoring at or above the previously established cut-point. This was established using receiver operator characteristic analyses to determine the best cut-point from the values of sensitivity and specificity calculated at increasing test score cut-points. This analysis yielded a score of 12 or more as indicative of competence at delivering CBT-E (area under the curve 0.964, sensitivity 0.909, specificity 0.881). Three equivalent versions of the measure are available so that different versions can be used on different assessment occasions.

### Randomization

An independent statistician, not otherwise involved in the study, randomized participants to independent and supported training. To ensure that therapists who worked in the same organization did not receive different forms of training, the trainees were randomized by zip code. The first therapist from each zip code was randomized to a training group thereby determining the assignment of the cluster, with further participants from that zip code being allocated to the same group. Minimization on size of cluster was used to balance the number of participants in each training arm.

### Data Analysis

To investigate the immediate and longer term effects of training, a mixed effects model was fitted to the scores from the competence measure. The use of a mixed effects model allowed all time points to be modeled simultaneously and all randomized therapists to be included in the model in an intent-to-train analysis. The model assumes that the data are missing at random conditional on the covariates included in the model and scores at the other time points. The model included separate fixed effects for the mean score and time by training group interactions at the posttraining and 6-month time points. This allowed the means in the independent and supported groups to vary both at posttraining and 6 months. The model also included a normally distributed random effect for person to account for repeated measures nested within a normally distributed random effect for zip code.

A second mixed effects model without training group effects was used to look at the change in scores over time in both the independent and supported groups combined.

Logistic regression, with adjustment for pretraining score, was used to assess scoring over the competence cut-point with a binary variable created to indicate a score above the previously determined competence cut-point. A clustered sandwich estimator to account for clustering within zip code was used.

Missing competence data were tabulated and a sensitivity analysis was carried out to assess their impact on the findings. This analysis examined what difference would be required between the means in those missing and those observed for there to be a statistically significant difference between the training groups and compared these to the differences in expected scores between the 2 groups. All analyses were conducted in Stata 14 (StataCorp LLC) .

## Results

### Recruitment

**Figure 1:**
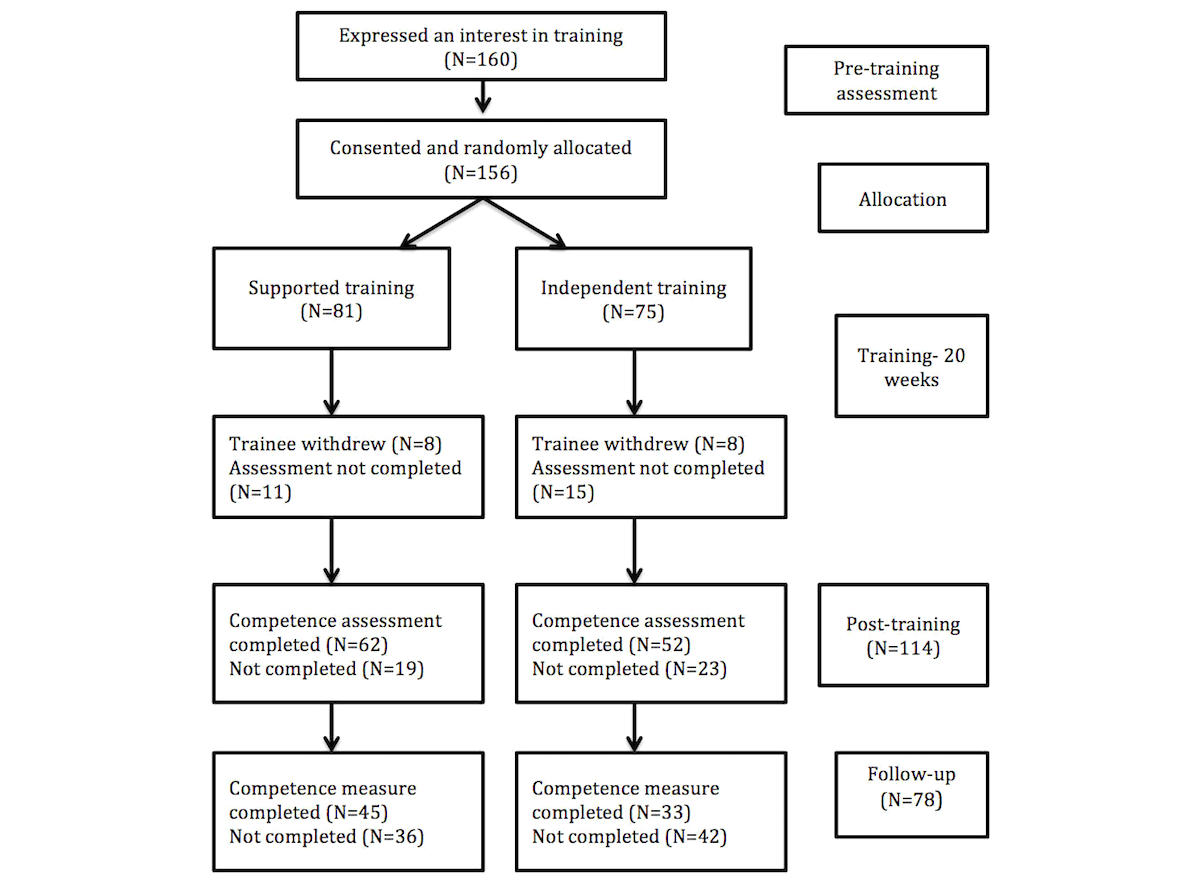
Flow of the participants in the study.

A total of 160 therapists expressed an interest in participating in the study, 156 (97.5%) of whom were randomized to the 2 forms of training (81 to supported training and 75 to independent training). These therapists were located in 30 different US states and 5 Canadian provinces. Figure 1 shows their progress through the study.

The median age of the participants was 36 years (interquartile range [IQR] 31 to 47; range 23 to 70 years) and 93.3% were female (140/150). They belonged to 2 main professional groups: 64 (41.0%) were clinical psychologists, while 45 (28.9%) were social workers. The remainder came from a variety of other backgrounds including counseling, family therapy, psychiatry, and psychiatric nursing. Median years of full-time equivalent clinical experience was 5.9 years (IQR 3 to 13.3; range 0 to 36 years). Participants reported seeing patients with eating disorders for face-to face treatment for a median of 14 hours per week (IQR 6 to 20; range 0 to 50 hours). Participant details by randomization group at baseline are shown in Table 1.

### Training Completion

The median number of modules of the training program completed was 14 out of a possible 18 (IQR 4 to 18). There was no strong evidence of a difference between the 2 training conditions in this regard (independent training 14 [IQR 1 to 18], supported training 16 [IQR 7 to 18], *P*=.10 [Somers D, adjusting for clustering by zip code]). The median number of support sessions received by those in the supported group was 10 (IQR 5 to 11).

### Immediate Effects of Training

**Figure 2:**
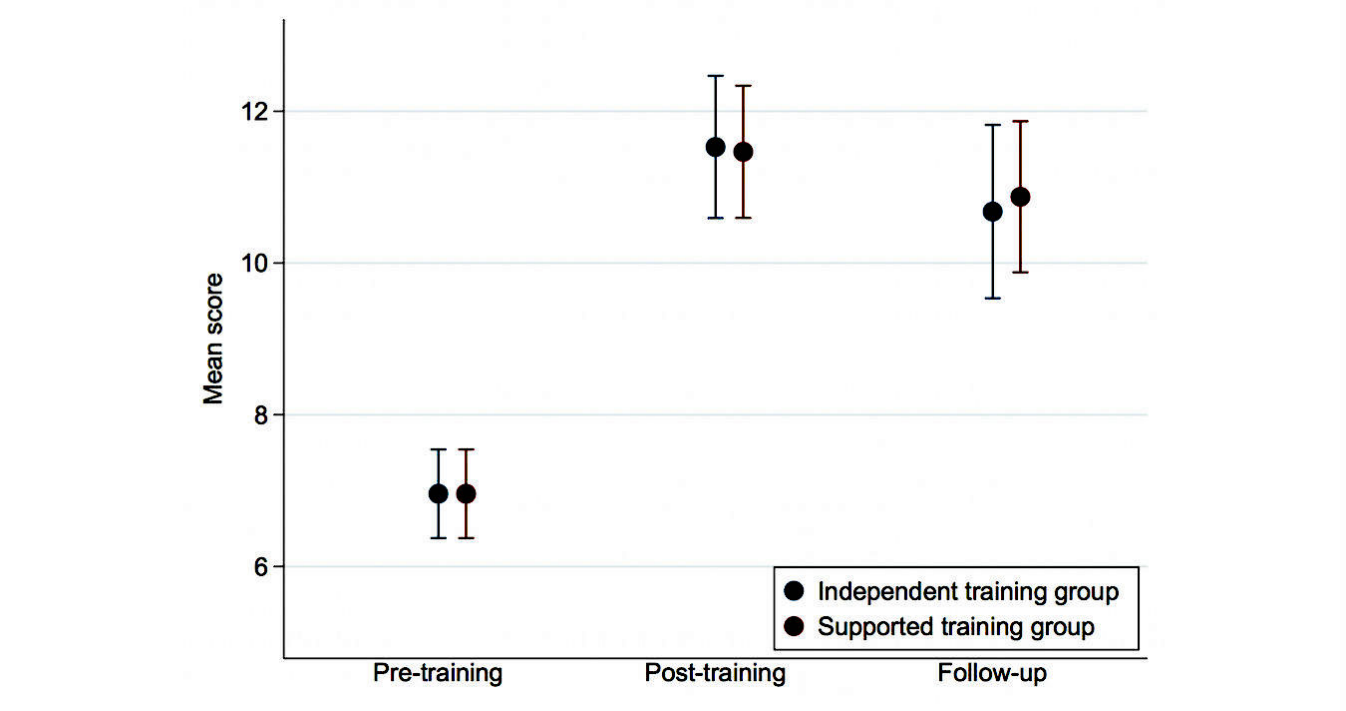
Mean scores and confidence intervals estimated by the mixed effects model for the 2 training groups.

Mean scores for the participants in the 2 training groups at the 3 assessment points are given in Table 2. The mixed effects analysis showed that there was an increase in competence scores in both training groups. Mean change score in the independent training group was 4.57 (95% CI 3.61 to 5.53; *P*<.001) while mean change in the supported training group was 4.51 (95% Cl 3.62 to 5.40; *P*<.001). As can be seen in Figure 2 there was no difference between the 2 forms of training in their effects on competence scores, with the mean difference for the supported versus independent group being –0.06 (95% Cl –1.29 to 1.16, *P*=.92).

After training, half of the participants (58/114, 50.9%) scored above the competence threshold. Of the 96 participants who had not met this threshold before training, 43 (43/96, 45%) met the threshold after training. Only 3 participants who had scored above the competence threshold before training failed to do so after training. The supported trainees were just as likely to score above the competence threshold as the independent trainees (odds ratio [OR] 1.02, 95% CI 0.52 to 1.99; *P*=.96).

### Effects of Training at Follow-Up

There was little change in participant competence scores from the end of training to the 6-month follow-up assessment (see Table 2). At follow-up assessment (see Figure 2), mean change in competence scores from pretraining baseline was 3.72 (95% Cl 2.56 to 4.88; *P*<.001) in the independent training group and 3.91 (95% Cl 2.90 to 4.93; *P*<.001) in the supported group. Again, the difference between the 2 forms of training was very small, with the mean difference for supported versus independent group being 0.19 (95% Cl –1.27 to 1.66, *P*=.80).

Table 2 Competence scores for the 2 training groups before and after training and at follow-up.

**Table 1:** Baseline characteristics of randomized therapists by training group.

Characteristics		Supported training (n=81)	Independent training (n=75)
Age, years, median (IQR^a^)		36 (29-45)	36 (32-47)
Female^b^, n (%)		75 (94)	65 (93)
**Professional background, n (%)**			
	Clinical psychologist	35 (43)	29 (39)
	Social worker	23 (28)	22 (29)
Weekly contact hours with patients with eating disorders^c^, median (IQR)		12.5 (6.0-18.5)	15.0 (7.0-21.0)
Clinical experience, full-time equivalent years^d^, median (IQR)		4.7 (2.6-13.0)	6.0 (3.5-14.0)

^a^IQR: interquartile range.

^b^Data missing for 1 participant in the supported training group and 5 in the independent group.

^c^Data missing for 1 participant in the independent group.

^d^Data missing for 3 participants in the supported group and 1 participant in the independent group.

**Table 2:** Competence scores for the 2 training groups before and after training and at follow-up.

Time point		Independent training	Supported training	All participants
**Pretraining**				
	Number of participants	75	81	156
	Mean competence score (range)	6.6 (1-19)	7.3 (2-16)	7.0 (1-19)
	Number competent, n (%)	9 (12)	11 (14)	20 (13)
**Posttraining**				
	Number of participants (% completing competence assessment)	52 (69)	62 (77)	114 (73)
	Mean competence score (range)	11.6 (0-18)	11.7 (4-19)	11.7 (0-19)
	Number competent, n (%)	26 (50)	32 (52)	58 (51)
**Follow-up**				
	Number of participants (% completing competence assessment)	33 (44)	45 (56)	78 (50)
	Mean competence score (range)	11.0 (3-18)	11.2 (2-17)	11.1 (2-18)
	Number competent, n (%)	16 (48)	21 (47)	37 (47)

Results from the second mixed effects model without the effects for training group indicated that although scores decreased over time, the change from end of training to follow-up was not statistically significant (mean difference –0.70, 95% CI –1.52 to 0.11; *P*=.09). There was no difference between the 2 training groups in the odds of scoring over the competence threshold (OR 0.95, 95% Cl 0.34 to 2.62; *P*=.92).

### Sensitivity Analysis

The sensitivity analysis suggested that unless fairly extreme assumptions were adopted about the differences between the mean scores of those whose scores are missing as compared to those observed, the present findings are relatively robust. (see Multimedia Appendix 2).

## Discussion

### Principal Findings

The findings of this study comparing 2 forms of Web-centered therapist training replicate and extend those of the earlier proof-of-concept study [13]. They show that Web-centered training is acceptable to therapists and that it is effective. The great majority of the training modules were completed, and scores on the competence measure increased significantly. As in the proof-of-concept study of the supported form of training, almost half the participants obtained competence scores indicative of a good level of competence, and this was true of both the supported and independent forms of training. Furthermore, the changes obtained with both forms of training appeared to be well maintained.

### Comparisons With Other Studies

There has been limited research on the outcome of training against which to compare the present findings [6,11,21]. Therapist training in general has been relatively neglected as a research topic until recently [22,23], and few studies have used validated competence measures [24]. Competence figures that have been reported following training in psychological treatment for depression range from 21% after attending a training workshop to 96% after extensive consultation with an expert including session review and feedback [25]. A study of community clinicians receiving training in transdiagnostic cognitive behavior therapy reported 59.5% of clinicians competent after training [26]. However, the latter training also involved extensive expert consultation and session review of a kind that is not scalable; thus, the findings are not directly comparable. Last, as part of our ongoing training program we have collected data employing the same competence measure as used in this study with therapists undergoing conventional training. Training involved an expert-led face-to-face workshop and 20 sessions of expert supervision while treating patients. Of 20 therapists studied to date, 19 were not competent at the start of training, and at completion of training 10 were competent (53%). As these trainees received extensive expert supervision, the findings are not directly comparable to those obtained in this study.

### Study Strengths

The study has a number of strengths. First, a relatively large number of trainees, dispersed across an extensive geographical area, was recruited and trained. Second, the trainees were randomized to 2 scalable forms of training thereby adding to the limited literature on scalable therapist training. Third, it used a validated measure of therapist competence that had an empirically established competence threshold that distinguished between therapists independently judged to be competent and those who were not. Fourth, the study included a follow-up assessment to investigate the durability of training effects.

### Study Limitations

The study also has limitations. First, it did not include a no-training control condition or a delayed training group. Thus, we cannot discount the possibility that competence scores would have increased over time without training, but this seems unlikely. Second, there was a significant amount of missing data, especially at the follow-up assessment 6 months after training. While this is clearly regrettable, the results of the sensitivity analyses indicate that this was unlikely to distort the overall findings. Attrition, both in the form of participants ceasing to use an intervention and not completing study assessments, has been noted as a particular problem in Internet interventions [27]. Third, the sample was not sufficiently large to investigate the characteristics of those who do and do not benefit from this form of training—for example, whether there are gender differences in the uptake and outcome of training.

### Conclusions

This study confirms that Web-centered training can successfully train a large number of therapists dispersed across a wide geographical area. Training on this scale cannot be provided with current methods of training. Another striking finding is that the training was equally effective whether undertaken independently or accompanied by support. Given the high degree of scalability of independent training, this finding is of great practical importance.

Independent Web-centered training therefore provides a means of training large numbers of geographically dispersed therapists at low cost, overcoming several obstacles to the dissemination of psychological treatments.

## Appendix

Multimedia Appendix 1 Enhanced cognitive behavioral therapy training program.

Multimedia Appendix 2 Sensitivity analysis.

## Abbreviations

CBT-E: enhanced cognitive behavioral therapy
IQR: interquartile range
OR: odds ratio

## References

[1] Kazdin AE (2017). Addressing the treatment gap: a key challenge for extending evidence-based psychosocial interventions. Behav Res Ther.

[2] Fairburn CG, Patel V (2014). The global dissemination of psychological treatments: a road map for research and practice. Am J Psychiatry.

[3] Kazdin AE, Fitzsimmons-Craft EE, Wilfley DE (2017). Addressing critical gaps in the treatment of eating disorders. Int J Eat Disord.

[4] Herschell AD, Kolko DJ, Baumann BL, Davis AC (2010). The role of therapist training in the implementation of psychosocial treatments: a review and critique with recommendations. Clin Psychol Rev.

[5] Fairburn CG, Cooper Z (2011). Therapist competence, therapy quality, and therapist training. Behav Res Ther.

[6] Martino S, Paris M, Añez L, Nich C, Canning-Ball M, Hunkele K, Olmstead TA, Carroll KM (2016). The Effectiveness and cost of clinical supervision for motivational interviewing: a randomized controlled trial. J Subst Abuse Treat.

[7] Zandberg LJ, Wilson GT (2013). Train-the-trainer: implementation of cognitive behavioural guided self-help for recurrent binge eating in a naturalistic setting. Eur Eat Disord Rev.

[8] Ray ML, Wilson MM, Wandersman A, Meyers DC, Katz J (2012). Using a training-of-trainers approach and proactive technical assistance to bring evidence based programs to scale: an operationalization of the interactive systems framework's support system. Am J Community Psychol.

[9] Fairburn CG, Wilson GT (2013). The dissemination and implementation of psychological treatments: problems and solutions. Int J Eat Disord.

[10] Khanna MS, Kendall PC (2015). Bringing technology to training: Web-based therapist training to promote the development of competent cognitive-behavioral therapists. Cogn Behav Pract.

[11] Waller G, Turner H (2016). Therapist drift redux: why well-meaning clinicians fail to deliver evidence-based therapy, and how to get back on track. Behav Res Ther.

[12] Cooper Z, Bailey-Straebler Suzanne (2015). Disseminating evidence-based psychological treatments for eating disorders. Curr Psychiatry Rep.

[13] Fairburn CG, Allen E, Bailey-Straebler S, O'Connor ME, Cooper Z (2017). Scaling up psychological treatments: a countrywide test of the online training of therapists. J Med Internet Res.

[14] Andrews G, Williams AD (2014). Up-scaling clinician assisted internet cognitive behavioural therapy (iCBT) for depression: a model for dissemination into primary care. Clin Psychol Rev.

[15] Traviss-Turner GD, West RM, Hill AJ (2017). Guided self-help for eating disorders: a systematic review and metaregression. Eur Eat Disord Rev.

[16] Wilson GT, Zandberg LJ (2012). Cognitive-behavioral guided self-help for eating disorders: effectiveness and scalability. Clin Psychol Rev.

[17] Fairburn C (2008). Cognitive Behavior Therapy and Eating Disorders.

[18] National Institute for HealthCare Excellence (2017). Eating Disorders: Recognition, Assessment and Treatment.

[19] Fairburn C, Cooper Z, Shafran R, Bohn K, Hawker D, Murphy R, Straebler S, Fairburn CG (2008). Enhanced cognitive behavior therapy for eating disorders: the core protocol. Cognitive Behavior Therapy and Eating Disorders.

[20] Cooper Z, Doll H, Bailey-Straebler S, Kluczniok D, Murphy R, O'Connor ME, Fairburn CG (2015). The development of an online measure of therapist competence. Behav Res Ther.

[21] Waller G (2016). Treatment protocols for eating disorders: clinicians' attitudes, concerns, adherence and difficulties delivering evidence-based psychological interventions. Curr Psychiatry Rep.

[22] Rosen RC, Ruzek JI, Karlin BE (2017). Evidence-based training in the era of evidence-based practice: challenges and opportunities for training of PTSD providers. Behav Res Ther.

[23] Harvey AG, Gumport NB (2015). Evidence-based psychological treatments for mental disorders: modifiable barriers to access and possible solutions. Behav Res Ther.

[24] McHugh RK, Barlow DH (2010). The dissemination and implementation of evidence-based psychological treatments: a review of current efforts. Am Psychol.

[25] Walser RD, Karlin BE, Trockel M, Mazina B, Barr-Taylor C (2013). Training in and implementation of Acceptance and Commitment Therapy for depression in the Veterans Health Administration: therapist and patient outcomes. Behav Res Ther.

[26] Creed TA, Frankel SA, German RE, Green KL, Jager-Hyman S, Taylor KP, Adler AD, Wolk CB, Stirman SW, Waltman SH, Williston MA, Sherrill R, Evans AC, Beck AT (2016). Implementation of transdiagnostic cognitive therapy in community behavioral health: the Beck Community Initiative. J Consult Clin Psychol.

[27] Eysenbach G (2005). The law of attrition. J Med Internet Res.

