# Supplementary material for: Using the Internet to Train Therapists: Randomized Comparison of Two Scalable Methods
Source: J Med Internet Res. 2017 Oct 18;19(10):e355. doi: 10.2196/jmir.8336 (PMC5666223; doi:10.2196/jmir.8336)
Supplement: Multimedia Appendix 1 [file jmir_v19i10e355_app1.pdf]

Multimedia Appendix 1- CBT-E Training programme

This web-based CBT-E training programme is designed to help clinicians implement CBT-E well. It centres on a large collection of video presentations by an expert on the treatment, and it is in three sections, **The Introduction**, **The Course** and **The Library**. The Introduction is a brief account of the nature and style of CBT-E. The Course is a detailed and practical description of how to implement CBT-E. It is linear in its organisation and additive in nature. It is therefore important that The Course is followed in the order in which the material is presented. In contrast, The Library is a repository of training material that can be accessed at any time.

The Introduction

The Introduction is a brief overview of CBT-E lasting about 40 minutes. It addresses the following topics:

- The indications for CBT-E and its goals
- The formulation (case conceptualisation)
- The duration of CBT-E and its two forms
- The style of CBT-E
- Preparing patients for CBT-E and implementing it well
- The structure and content of CBT-E

The Course

The goal of The Course is to help clinicians implement the focused form of CBT-E (20-session version). The four stages of CBT-E, and their component modules, are described in turn with each one being the focus of one or more tutorials. Most of the tutorials last between 20 and 30 minutes and each comprises a video-based description of the topic concerned accompanied by clinical illustrations, learning exercises and handouts in the form of downloadable PDFs. The total length of The Course is about nine hours. The table below lists the tutorials in The Course together with their duration.

The Library

The Library is a resource to be consulted at any time. It contains the following material:

1. All the material in The Course
2. Additional modules, including ones on the following topics:
    - Younger Patients
    - Underweight patients (anorexia nervosa)
    - Mood intolerance
    - Broad form of CBT-E
        - Clinical perfectionism
        - Core low self-esteem
        - Marked interpersonal problems
3. A large number of additional clinical demonstrations. These are longer than those embedded within The Course.

The Library is indexed to facilitate searching.

**WEB-CENTRED TRAINING IN CBT-E**<br>**Content and Timings**	
**THE INTRODUCTION (about 1 hour)**	
Overview of the training website	10 mins
Overview of the treatment	40 mins
**THE COURSE (about 9 hours in total)**<br><br>**Stage One (2 hrs 40 mins)**	
Starting Well Module	
The initial session	40 mins
Creating a formulation	20 mins
Establishing self-monitoring	30 mins
Weight, weighing and weight concern	40 mins
Establishing regular eating	30 mins
**Stage Two (40 mins)**	
Taking Stock Module	
Reviewing progress	20 mins
Planning Stage Three	20 mins
**Stage Three (4 hrs 20 mins)**	
Body Image Module	
Over-evaluation of shape and weight	40 mins
Developing other domains	15 mins
Body checking	35 mins
Comparison-making	20 mins
Body avoidance	20 mins
Feeling fat	25 mins
Events, Moods and Eating Module	25 mins
Dietary Restraint Module	50 mins
Setbacks and Mindsets Module	30 mins
**Stage Four and the Post-Treatment Review Session (1 hour)**	
Ending Well Module	30 mins
Post-treatment Review Session	30 mins
