# Supplementary material for: Using the Internet to Train Therapists: Randomized Comparison of Two Scalable Methods
Source: J Med Internet Res. 2017 Oct 18;19(10):e355. doi: 10.2196/jmir.8336 (PMC5666223; doi:10.2196/jmir.8336)
Supplement: Multimedia Appendix 2 [file jmir_v19i10e355_app2.pdf]

Multimedia Appendix 2- Sensitivity analysis

Sensitivity analyses were conducted using rctmiss, a Stata package for sensitivity analyses for randomised trials analysis [1]. Sensitivity analyses were conducted on two separate sets of linear regression models: for change post-training and for change at 6-months, both adjusted for pre-training score and accounting for zip-code area.

The sensitivity analyses examined the size of the difference required between the means in those missing and those observed for a statistically significant difference between the training groups. Four scenarios were considered for each model:

1. Missing participants in the independent training group had the same mean score as those who were not missing and those missing in the supported group had a higher mean score than those not missing
2. Missing participants in the supported training group had the same mean score as those who were not missing and those missing in the independent training group had a higher mean score than those who were not missing
3. Missing participants in the independent training group had the same mean score as those not missing and those missing in the supported group had a lower mean score than those not missing
4. Those missing in the supported training group had the same mean score as those not missing and those missing in the independent group had a lower mean score than those not missing

The results of the sensitivity analysis for change in competence score immediately after training are given in Table A1. As can be seen the mean score of missing participants would have to be substantially different (more than 5 points) from those observed in all scenarios to produce a statistically significant difference between the training groups.

Table A1 Sensitivity analysis for change in competence score after training.

Scenarios for missing scores	Difference in mean score in missing compared to observed for $p<0.05$	Difference in expected score in supported group compared to independent group for the scenario (95% CI; p-value)
Independent group mean equal to observed & supported group > mean (scenario 1)	6.39	1.54 (0.00 to 3.07; 0.049)
Supported group mean equal to observed & independent group > mean (scenario 2)	5.14	-1.52 (-3.03 to -0.00; 0.049)
Independent group equal mean to observed & supported group < mean (scenario 3)	-6.42	-1.54 (-3.07 to -0.00; 0.049)
Supported group mean equal to observed & independent group < mean (scenario 4)	-5.11	1.51 (0.00 to 3.03; 0.049)

.

Results of the sensitivity analysis for change in score at 6-month follow up are given in Table A2. In this case the mean score of those missing would have had to be different by more than 2 points from those observed in their training group for a statistically significant difference between groups.

Table A2 Sensitivity analysis for change in competence score at 6-month follow up.

Scenario	Difference in mean score in missing compared to observed for p<0.05 (points)	Difference in expected score in supported group compared to independent group for the scenario (95% CI; p-value)
Independent group mean equal to observed & supported group > mean (scenario 1)	2.94	1.61 (0.01 to 3.21; 0.049)
Supported group mean equal to observed & independent group > mean (scenario 2)	3.48	-1.61 (-3.22 to -0.01; 0.049)
Independent group equal mean to observed & supported group < mean (scenario 3)	-4.44	-1.68 (-3.35 to -0.00; 0.049)
Supported group mean equal to observed & independent group < mean (scenario 4)	-2.33	1.58 (0.01 to 3.16; 0.049)

1. White IR, Carpenter J, Horton NJ. A mean score method for sensitivity analysis to departures from the missing at random assumption in randomised trials. arXiv:1705.00951v1
